# Extraction of Value-Added Minerals from Various Agricultural, Industrial and Domestic Wastes

**DOI:** 10.3390/ma14216333

**Published:** 2021-10-23

**Authors:** Virendra Kumar Yadav, Krishna Kumar Yadav, Vineet Tirth, Govindhan Gnanamoorthy, Nitin Gupta, Ali Algahtani, Saiful Islam, Nisha Choudhary, Shreya Modi, Byong-Hun Jeon

**Affiliations:** 1 Department of Microbiology, School of Sciences, P P Savani University, Kosamba, Surat 394125, Gujarat, India; yadava94@gmail.com; 2 Faculty of Science and Technology, Madhyanchal Professional University, Ratibad, Bhopal 462044, India; envirokrishna@gmail.com; 3 Mechanical Engineering Department, College of Engineering, King Khalid University, Abha 61411, Asir, Saudi Arabia; vtirth@kku.edu.sa (V.T.); alialgahtani@kku.edu.sa (A.A.); 4 Research Center for Advanced Materials Science (RCAMS), King Khalid University, Guraiger, Abha 61413, Asir, Saudi Arabia; 5 Department of Inorganic Chemistry, University of Madras, Chennai 660025, Tamil Nadu, India; gnanadrdo@gmail.com; 6 School of Nanosciences, Central University of Gujarat, Gandhinagar 382030, Gujarat, India; nitinkgupta1988@gmail.com (N.G.); nishanaseer03@gmail.com (N.C.); 7 Civil Engineering Department, College of Engineering, King Khalid University, Abha 61413, Asir, Saudi Arabia; sfakrul@kku.edu.sa; 8 Department of microbiology, Shri Sarvajanik Science College, Mehsana 384001, Gujarat, India; shreyamodi20@gmail.com; 9 Department of Earth Resources and Environmental Engineering, Hanyang University, Seoul 04763, Korea

**Keywords:** waste, agricultural waste, value-added materials, calcium oxide, eggshell, incense sticks

## Abstract

Environmental pollution is one of the major concerns throughout the world. The rise of industrialization has increased the generation of waste materials, causing environmental degradation and threat to the health of living beings. To overcome this problem and effectively handle waste materials, proper management skills are required. Waste as a whole is not only waste, but it also holds various valuable materials that can be used again. Such useful materials or elements need to be segregated and recovered using sustainable recovery methods. Agricultural waste, industrial waste, and household waste have the potential to generate different value-added products. More specifically, the industrial waste like fly ash, gypsum waste, and red mud can be used for the recovery of alumina, silica, and zeolites. While agricultural waste like rice husks, sugarcane bagasse, and coconut shells can be used for recovery of silica, calcium, and carbon materials. In addition, domestic waste like incense stick ash and eggshell waste that is rich in calcium can be used for the recovery of calcium-related products. In agricultural, industrial, and domestic sectors, several raw materials are used; therefore, it is of high economic interest to recover valuable minerals and to process them and convert them into merchandisable products. This will not only decrease environmental pollution, it will also provide an environmentally friendly and cost-effective approach for materials synthesis. These value-added materials can be used for medicine, cosmetics, electronics, catalysis, and environmental cleanup.

## 1. Introduction

Every day we come across various types of waste in our life either in houses, working areas, industries, farms, etc. These waste materials can be kitchen waste, agricultural waste, industrial waste, or poultry waste. Although there are also several other types of waste, they are outside the scope of the present study. The waste produced in our houses, such as kitchen waste and incense stick ash can be categorized as household waste [1], whereas the waste produced from the agricultural practices, such as wheat straw [2,3], wheat husks [4], rice straw [5], rice husks [6], coconut shells [7], palms and dates [8], lemon peel [9], almond shells [10], etc., can be considered to be agricultural waste [11]. The waste generated by industry, such as coal fly ash (CFA) [12,13], red mud [14,15], gypsum waste [16,17], sewage sludge [18], iron tailing [19], etc., fall under the category of industrial waste [20]. Eggshells [21,22,23] and poultry litter [24,25] can be categorized as poultry waste; however, most investigators have considered eggshells to also belong to industrial waste. Among the above-mentioned types of waste, agricultural and industrial wastes raise major concerns for the environment, as millions of tons are produced every year around the world. Most of these waste materials are still disposed of via landfill [26,27], dumping, or disposal in water, creating pollution of the environment. The dumping of such waste may further deteriorate fertile agricultural soil [28], produce a foul odor [29] that may attract pests and mosquitos, and lead to health issues for human beings and animals [30]. Some types of industrial waste, like CFA [31] and red mud [32], are considered hazardous because of their high concentration of toxic heavy metals [33]. Heavy metals from dumping areas can leach into the surrounding soil, and when it rains, they may percolate into water bodies, thus leading to water pollution [34].

Nowadays, with the advent of new technologies, such waste can be processed into value-added minerals, especially in metallurgy. They can also be applied in the fields of agriculture [35], adsorbents [36,37], geo-polymers [38,39], ceramics [40], and environmental cleanup [41]. This article reviews and discusses a variety of waste materials in detail, including silica, alumina, calcium oxides, carbonates, etc., along with their properties, applications, and methods for their recovery. 

## 2. Industrial Waste

**Figure 1:**
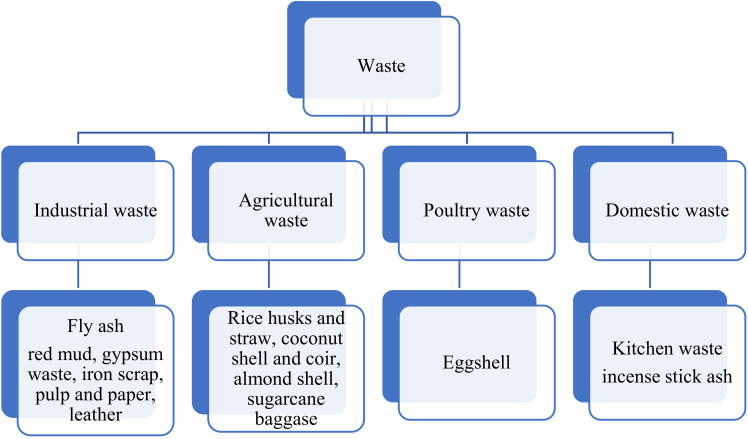
Classifications of the different valuable waste materials.

Waste can be classified into several classes depending on its origin, for instance, industrial waste (CFA, paper and pulp waste), agricultural waste (coconut coir, sugarcane bagasse, and lemon peel) [42,43,44,45,46,47,48,49]. Similarly, waste materials produced in the home are referred to as domestic waste, including kitchen waste and incense stick ash, while eggshells and waste from poultry are regarded as poultry waste. The major categories of waste are shown below in Figure 1.

Industrial waste refers to byproducts, e.g., CFA, red mud, gypsum waste, and other municipal waste, generated in the industrial sector. Among the various types of waste, this is one of the major sources that requires special attention due to its hazardous nature.

### 2.1. Fly Ash

Coal fly ash consists of fine powders produced from the pulverized coal used in the thermal power plants during the generation of electricity. It is heterogeneous in nature, and is made up of a glassy amorphous phase and a crystalline quartz phase, such as mullite and magnetite. Fly ash is produced in large amounts in TPPs, and is mainly considered waste, although its mineral value makes it a useable material.

#### 2.1.1. Recovery of Minerals from CFA

CFA can be applied in several ways in the fields of agriculture, remediation (as an economical adsorbent), ceramics, etc. [50]. Formerly, CFA was considered a hazardous waste [51], but today, it is considered to be a useable material. CFA has high amounts of micro- and macro-nutrients that can be used as a source of nutrients for plants [52,53]. It is used in agriculture (as a fertilizer) [54], forestation [55], reclamation of wasteland [56], and for maintaining the pH of acidic soil [56]. CFA is also used as an adsorbent for the removal of pollutants—mainly dyes [57,58], pesticides [57,58], and heavy metals [59,60]—from wastewater. It is used for making nanocomposites [61] that are applicable in the defense industry [62,63] and the production of lightweight materials [64,65]. CFA can be used for making blended cement [66] tiles, bricks [67], blocks [56], RCC, kitchen panels, and geo-polymers. CFA can also be used as fillers in rubbers and tires [68], and can also be used in the mining industry [56,69] for the recovery of ferrous metals [70], cenospheres [55], mullite [71], silica [72], zeolites [73], and alumina. It can also be applied for the recovery of unburnt carbon, soot, and carbon nanomaterials like carbon nanotubes, fullerenes, and graphene. These carbonaceous materials are formed due to the burning of the organic content of the coal, while soot and unburnt carbon are due to the incomplete burning of coal. Higher grades of coal like anthracite and bituminous coal have higher carbon contents, so after the burning of such coals in thermal power plants, the resulting ash will also have a high content of carbon in the form of unburnt carbon, soot, and carbon nanomaterials like carbon nanotubes, fullerene, etc.

##### Silica

**Figure 2:**
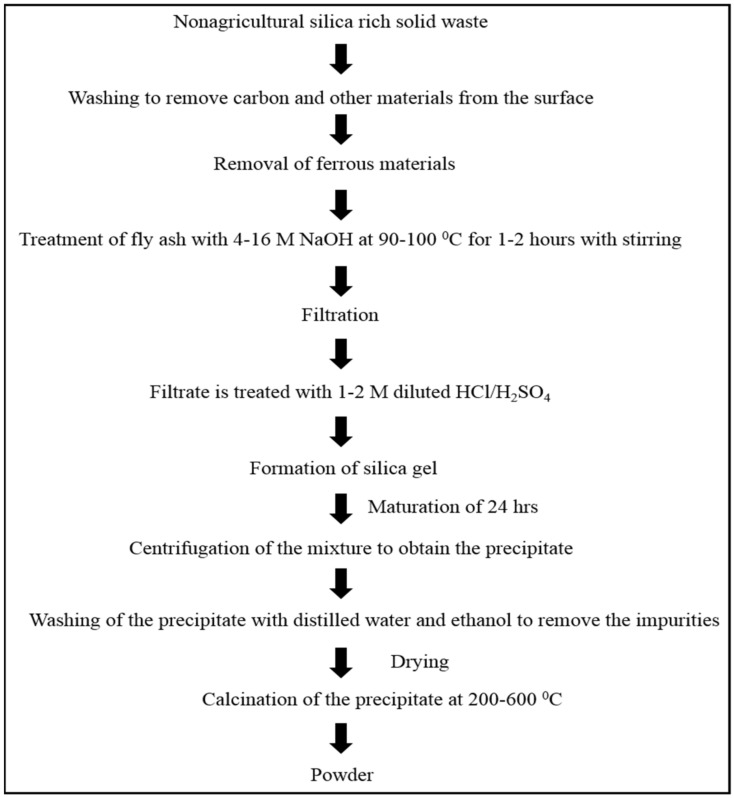
Schematic diagram of the synthesis of silica from silica-rich waste materials.

Silica accounts for up to 40–60% of CFA [74], depending on the source of the CFA, the type of coal, the operating parameters of the thermal power plant, the furnace temperature, etc. Silica is present in CFA in either crystalline or amorphous form [75]. The crystalline form of silica is present mainly as quartz, sillimanite, and mullite [76], whereas the glassy amorphous form is the only form of amorphous silica. Crystalline silica is mainly inert, and does not easily react with acids or bases. Therefore, silica can only be extracted from the amorphous form, as it reacts easily with strong bases. In addition, silica can easily be extracted from CFA through treatment with strong bases like NaOH, KOH, and sodium bicarbonate [77]. Silica is mainly extracted using either the alkali treatment [78] method or the alkali fusion method [79]. The complete extraction of silica using the alkali treatment method is depicted in Figure 2, while in alkali fusion method, the silica source is calcinated with NaOH at high temperature in order to form a new mineral (such as nephaline), from which it is easier and more efficient to extract the silica. Yadav et al. reported the extraction of nanosilica from fly ash tiles using the alkali fusion method [80]. Silica can be used in the production of glass and ceramics [80], in drug delivery [81], and used in medicine, foundries, adsorbents, catalytic processes [82], and molecular sieves [83]. Silica is nontoxic and mesoporous, meaning it can be readily used in various industries. The silica extracted from the CFA is nanosized (20–60 nm), but aggregates together to form lumps.

##### Alumina

Alumina, which is amphoteric in its nature, can be obtained from CFA or other alumina-rich materials using both acids and bases. Depending on the CFA type and the source of the coal, the alumina content in CFA can range between 20 and 40% [84]. In CFA, alumina can mostly be found in the form of crystalline aluminosilicates [85] such as mullite. These aluminates are highly inert, and rarely react with acids; thus, only low yields can be obtained. They can be treated with strong mineral acids that can be directly converted to powder by thermal decomposition. They can also be extracted using alkali like NaOH at high temperatures, i.e., 125–1100 °C. Alumina forms in numerous meta-stable phases, including gamma [γ]-, epsilon [η]-, delta [d]-, theta [θ]-, kappa [κ]- and *χ*-alumina. While the most stable phase of alumina is [*a*] alpha. Of all of the phases, the gamma-phase of alumina is the most important and widely used nanosized material. Gamma alumina is widely used in the petroleum and automobile industries as a catalyst and catalyst substrate, to produce ceramics [86] and glass, adsorbents, spacecraft materials, microelectronics, thermal-resistant materials, and as a coating material for thermal wear and abrasives [87], optoelectronics, and metallurgy. Alumina nanoparticles have high compression strength, high chemical resistance, a high degree of refractoriness, high thermal shock resistance, high abrasion strength, and high dielectric strength. Alumina is also applied in ceramics, adsorbents, fire-retardant materials, acid, and alkali-resistant materials [88].

##### Cenospheres

Cenospheres are aluminosilicate spheres [89,90], the size of which varies by microns. They are lightweight nanostructured materials that are created when coal is combusted within a furnace. These are used as lightweight materials for aircraft, in the field of defense, in fire-proof materials [55], and for acid and alkali wear-resistant materials [91]. Today, cenospheres that are tiny hollow spheres with diameter of approximately 10–1000 µm are among the most desirable byproducts that can be obtained from coal combustion processes. They normally comprise 1–2% of CFA obtained during the process of coal combustion. Cenospheres possess properties such as very high mechanical strength and low density; thus, they are regarded as a highly significant issue in coal-fired power plants. A number of parameters affect cenosphere properties, including the grinding operations, the nature of the coal used, the combustion parameters, and withdrawal when generating electricity. These materials are mainly possess a glassy surface and a crystalline matrix such as mullite with a nano-film covering with a thickness of 30–50 nm. Their form is similar to that of a shell, with a thickness varying between 2 and 30 microns. They are extracted using the density-based centrifugation method, during which finer lightweight cenospheres float at the top of the slurry, while the particles of heavier ferrous metals settle at the bottom [55]. The cenospheres are collected from the top and dried before use. The formation mechanism of cenospheres during pulverized coal combustion is complex and is highly dependent on fuel properties and combustion parameters. The formation mechanism of cenospheres is very similar to the procedure of glass blowing [92]. Therefore, it would be beneficial to take a closer look into glass formation principles.

The literature presents two approaches for the extraction of cenospheres from fly ash, namely dry separation and wet separation. The conventional cenosphere extraction method is primarily performed using wet processes, namely simple sedimentation and flotation [93]. There are two methods for estimating the degree of separation when recovering cenospheres from coal CFA, namely the float method and the sink method. To separate cenospheres from CFA, various liquids, viz., water (1 g/cc) and acetone (0.789 g/cc), can be used. Fly ash is kept in a vessel and water is added to it. The complete mass is stirred for four hours; afterwards, it is allowed to settle for ten hours. Then, all cenospheres with densities lower than 1 g/cc will float up and can be separated [94]. 

##### Mullite

Mullite, consisting of micron-sized particles (1–1.5 microns in length and 0.3–0.5 microns in width), is a rarely observed crystalline mineral that contains aluminum silicate (3Al_2_O_3_ 2SiO_2_) [95] and is mainly made up of Al, Si, and O; however, its composition can be quite variable. In the process of combusting aluminosilicate raw materials, mullite is created. This material is a key component in porcelains, ceramic whiteware, high temperature insulation, refractory materials and traditional ceramics [96]. Mullite is a compositional orthorhombic aluminosilicate, and generally possesses the composition Al_2_ (Al_2+2*x*_Si_2−2*x*_)O_10__−*x*_. Mullites are non-stoichiometric compounds whose structure is similar to magnetite containing impurities. It is rarely formed in nature, because of its formation conditions, which require high temperature in combination with low pressure. Synthesis of mullites is possible from high-silico-aluminous CFA only, as they are rich in silica and alumina, with lower contents of iron oxides. Iron oxides have a negative effect on mullite. Generally, for the purpose of synthesizing mullites from CFA, cenospheres are the most effective materials, since they have lower contents of iron oxides.

Mullites are formed in CFA from the organic and inorganic materials present in coal as a result of different melting and firing processes. After the extraction of mullites using a hydrofluoric process, it can be characterized by XRD and SEM-EDS in CFA. A clearer picture of mullite can be obtained using XRD and NMR. Both of these techniques can be used to efficiently investigate the value of x and the oxygen hole rate in the general formula, thus obtaining the mullite composition. Mullites can be used as refractory materials because of their high melting point (1840 °C).

Mullites are aluminosilicate minerals with the general formula Al_4+2x_ Si _2+2x_ O_10−x_ (with the value of x varying between 0.17 and 0.59). Mullites are capable of forming two stoichiometric forms, namely 3Al_2_O_3_2SiO_2_ and 2Al_2_O_3_ SiO_2_. Mullite is known to be the only stable binary phase of the A1_2_O_3_-SiO_2_ system that exists under ambient conditions. From an empirical perspective, its chemical compositions include 71.8 wt% A1_2_O_3_ and 28.2 wt% SiO_2_, designated as 3/2-mullite (3A1_2_Oy_2_SiO_2_). Mullite has two common morphologies: a platelet shape and a needle shape. In the platelet shape, it has a low aspect ratio, whereas in the needle shape, it possesses a high aspect ratio. In addition, mullite has low thermal conductivity, low thermal expansion, high thermal stability, high corrosion stability, high strength, and high fracture toughness. It has excellent creep resistance, acceptable thermal shock and stress resistance, and acceptable strength wear resistance, and it can be used at high temperatures. Mullites can be applied as an effective replacement for platinum in diesel engines, furnace liners, electrical insulators, protection tubes, kiln furniture, rollers, heat exchanger components, heat insulation parts, pressed parts, and isostatically pressed parts [97,98,99,100].

**Figure 3:**
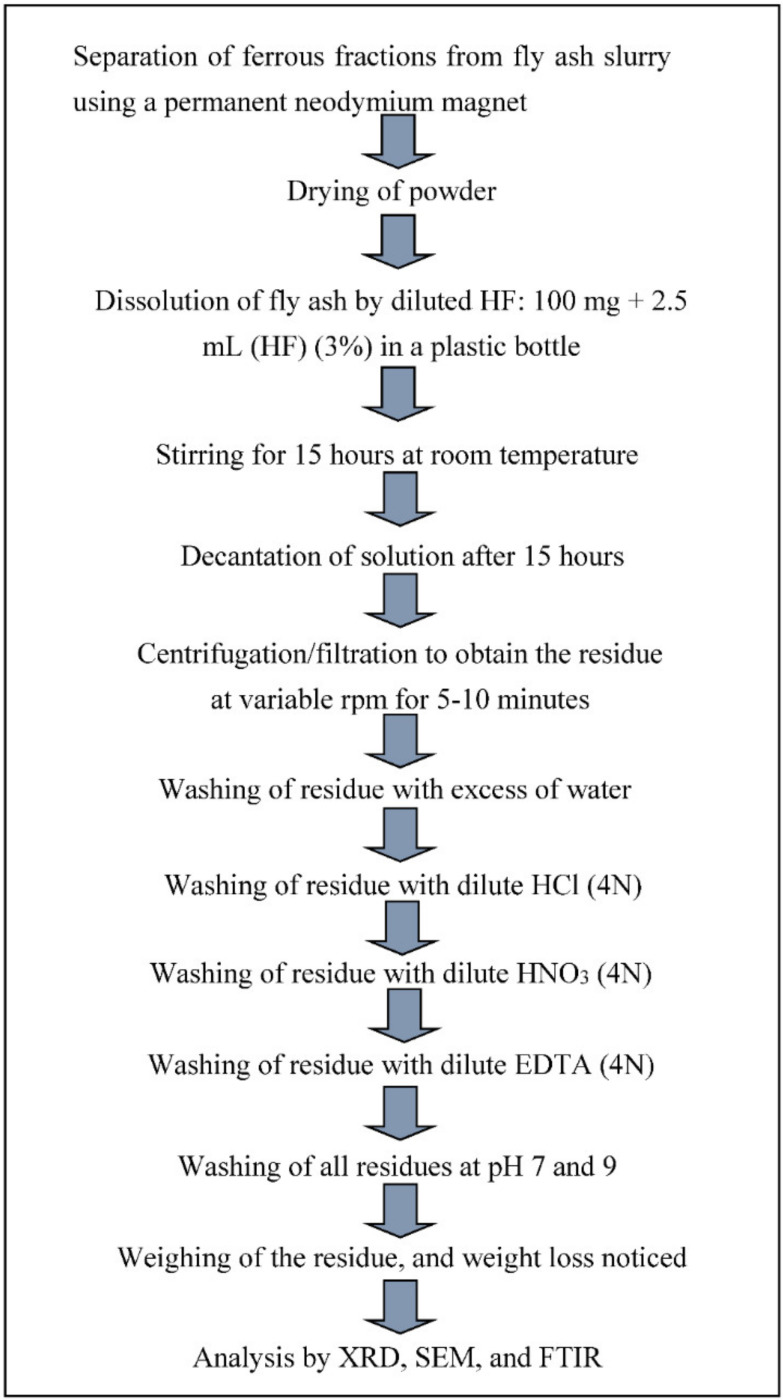
Schematic diagram for the recovery of mullite from CFA adopted from Yadav et al., open access journal Crystals, 2021 [94].

Yadav et al., 2021 reported the recovery of needle-shaped mullite, 90–300 nm in size, extracted from CFA using 16 M HF acid. An optimum ratio of CFA and HF was mixed and kept for interaction in an incubator shaker. The CFA was collected from the Gandhinagar and Gujarat thermal power plants. The source of the coal was anthracite/bituminous coal, i.e., higher grades of coal. The detailed mechanism for recovery of mullites from CFA is given below in Figure 3 [94].

##### Zeolites Synthesis from CFA

Zeolites are another class of materials that are widely used in industry for petroleum cracking, cation exchange resins, water softening processes, etc. These zeolites are also hydrates of alumina and silica, mainly containing Al, Si, cations from gp II (Ca, Mg, Na) and water molecules. Due to the presence of 40–60% silica and 20–40% alumina, the CFA serves as an economical resource for zeolite material synthesis. To date, zeolites have been synthesized from CFA by using 4–8 M, NaOH treatment along with continuous heating at 90–105 °C, for 1 h to 24 h with rigorous stirring. The pH, temperature, heating and stirring time decides the morphology and class of zeolites to be synthesized. There are several reports in the literature in which CFA from different parts of the globe has been used for the synthesis of zeolites. Yadav et al., 2019 and 2021, reported the synthesis of zeolites from CFA collected from the Gandhinagar and Gujarat TPPs in India. The size of the synthesized zeolites varied between 80 nm and 180 nm in width, while their length varied from 120 nm to 300 nm [99].

### 2.2. Red Mud

**Figure 4:**
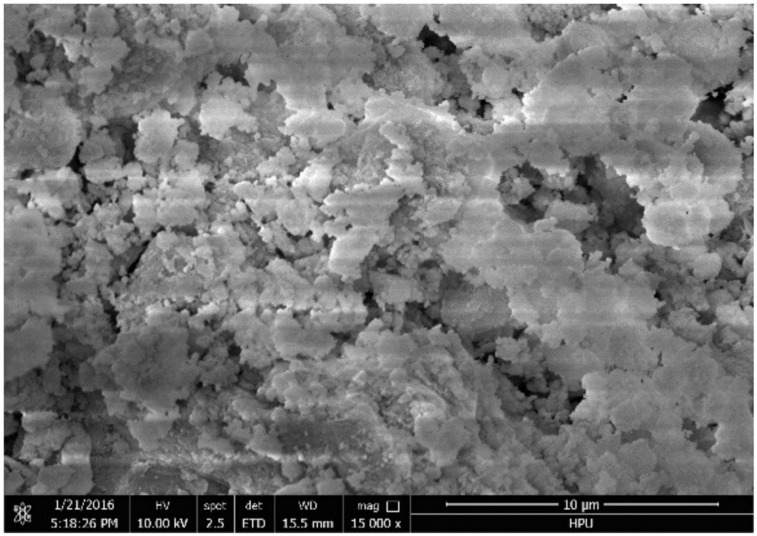
SEM micrograph of red mud, adopted from Zhang et al., open access journal Applied Sciences, 2018 [109].

Red mud is a hazardous byproduct of the bauxite industry [101], and is produced at the time of extraction of alumina from bauxite using Bayer’s method [102]. It is considered hazardous because of its high alkalinity and the presence of various toxic heavy metals; however, it is also a rich source of titanium, iron, and aluminum (composition is shown in Table 1). In addition, red mud is recognized as a bauxite residue [103], as it is what is left after the extraction of all the extractable alumina from bauxite. The resulting mud is a mixture of the insoluble fraction of solid and metallic oxides, and ore, which remains after the extraction of the aluminum-containing components [104]. It is typically disposed of as a slurry with a solid concentration ranging between 10 and 30%, pH in the range of 13, and high ionic strength [14]. This disposal problem is compounded by the fact that typical bauxite processing produces up to three times as much toxic red mud as aluminum. Figure 4 shows a typical SEM micrograph of red mud. The particles of red mud aggregate together to form lumps.

Approximately 44 million tons [MTs] of primary aluminum are produced annually around the world [105]; by that count, roughly 132 MTs of red mud enter retention ponds and some dry stack tailing areas annually [106]. The alumina plants in the Indian context have an annual capacity of 1.692 MTs; they produce 0.6 MTs of metal, and approximately 2 MTs of red mud per year [107]. The chemical analysis of RM reveals the presence of silica, aluminum, iron, calcium, and titanium, as well as an array of minor constituents, namely Na, K, Cr, V, Ni, Ba, Cu, Mn, Pb, Zn, etc. [15,108]. There is a high variance in chemical composition among various red muds generated around the world [14].

Currently, in the alumina industry, alumina is obtained by carrying out Bayer’s process on bauxite. The byproduct of such processes is red mud, which is generally regarded as dangerous waste, because it contains high concentrations of some heavy metals, iron oxide, and other metal oxides. Industry does not recycle it, and there is no available disposal method for it. Currently, companies collect this waste in their vicinity after the extraction of alumina. This continuous piling up of red mud constitutes a major and global threat to the environment [110,111]. This red mud contains high amounts of iron oxides and heavy metals, which can leach out into water bodies and cause damage to aquatic fauna. In India, two major alumina-producing companies are NALCO and HINDALCO, who generate a huge amount of waste every year in the form of red mud. This causes water pollution through its entry into water streams, leaching toxic heavy metals into water bodies. This also affects the aquatic flora and fauna. In addition, it has adverse effects on the biological oxygen demand (BOD) and chemical oxygen demand (COD) of the water bodies, resulting in a lowering of these parameters. This waste changes the community structure of water bodies. The alkalinity of water bodies is increased as a result of releasing NaOH into the water. In any alumina refinery, large amounts of land are required for the handling of this waste.

#### 2.2.1. Recovery of Alumina from Red Mud

**Figure 5:**
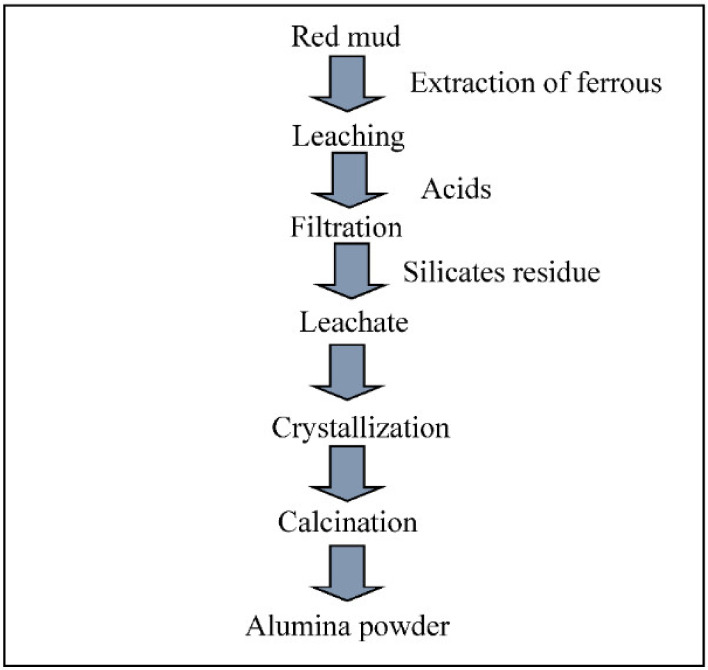
Flow chart for the recovery of alumina from red mud/CFA.

The red mud-based recovery of alumina involves several steps in series—recovery of ferrous particles, leaching of dried red mud with strong mineral acids, filtration to obtain the filtrate, crystallization of the leachate, the recovery of acids, and finally calcination—in order to obtain the alumina powder. A complete flow chart depicting the extraction of alumina from red mud is given below in Figure 5.

The recovery of alumina and ferric oxides from iron-rich red mud has been reported using the reduction sintering technique [112]. Different experiments have shown that up to 89.71% of alumina can be extracted, with a Fe recovery rate of 60.67%, under optimal conditions [113]. Zhang et al. [114] investigated andradite-grossular hydrogarnet formation in the hydrothermal process with the aim of examining its effect on alumina and alkali recovery from Bayer red mud. For the evaluation of the parameters with the highest impact on the recovery process, they took into consideration the batch experiments and parameters such as caustic ratio (molar ratio of Na_2_O to Al_2_O_3_ in sodium solution), reaction temperature, residence time, and sodium concentration. Zhu et al. [115] reported the recovery of alumina and alkali from red mud using a novel calcification–carbonation method under mild reaction conditions. Batch experiments were performed, and the effects of temperature, pressure, and additive addition on the extraction efficiency of alumina were examined, and the extraction efficiency of alumina was 95.2%. In another study, Meher [116] reported the extraction of alumina from red mud using a calcium carbonate and sodium carbonate sintering process. They studied the impacts of Na_2_CO_3_ and CaCO_3_ additives, sintering time and temperature, and leaching time on the effectiveness of alumina extracted from red mud. The alumina extraction was up to 97.64% at a sintering temperature of 1100 °C for 4 h with red mud.

#### 2.2.2. Applications of Red Mud

**Figure 6:**
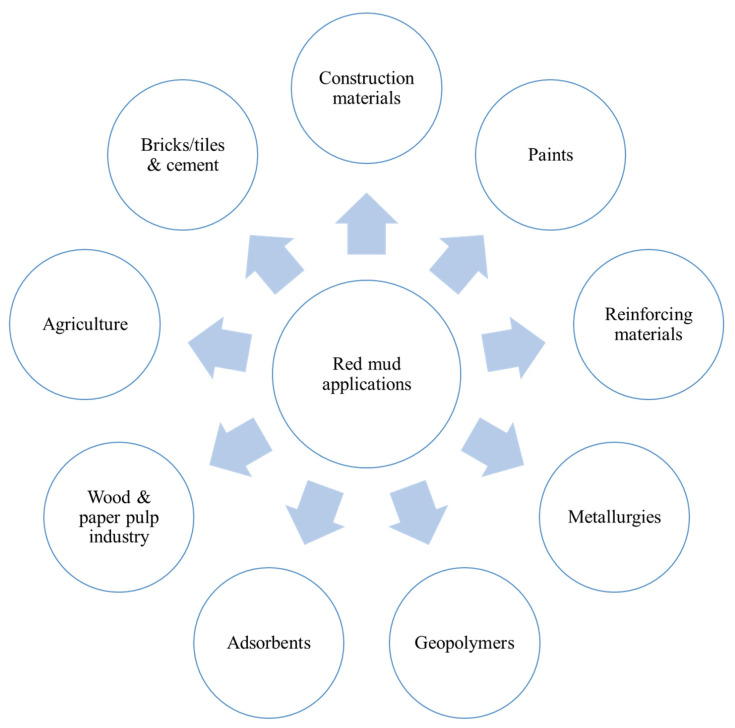
Diagrammatic representation of red mud in their broader applications.

Many studies have confirmed the benefits of red mud in the process of treating water and removing inorganic anions (e.g., fluoride, phosphate, and nitrate), toxic heavy metals and metalloid ions, as well as organic substances (e.g., phenolic compounds, dyes, and bacteria) [117,118]. Moreover, red mud can be employed as an effective catalyst in processes such as hydrocarbon oxidation, hydrodechlorination, and hydrogenation [119]. The broader areas of application of red mud are given in Figure 6.

### 2.3. Iron Slags/Scraps

There are several iron-based industries, like the steel industry, where iron particles are generated as waste materials [120]. These iron materials can be processed for the recovery of highly pure iron, zero-valent iron, or iron oxides. These can be used directly as filings or in coke industries [121]; thus, they have drawn much attention. These iron particles can be recovered using strong magnets in either wet or slurry form. These iron particles can further be treated with acids to obtain iron-rich leachates that can be used as precursor materials for the synthesis of different types of iron oxide particles. The synthesized iron oxide particles can be recovered by precipitation, chemical precipitation, and calcination. These iron oxides are so highly pure that they can act as an adsorbent in processes like the treatment of wastewater, environmental cleanup, in ceramics, and in the steel industry [122,123]. The major advantages of such iron oxide particles include their ability to be easily recovered from the reaction site, their recyclability, and their easy external manipulation using strong magnets [124]. The synthesis of iron oxide particles from waste is cost-effective and environmentally friendly.

Tang et al. [125] attempted a coal-based smelting reduction method for the recovery of Fe, Ni, and Cr from pickling sludge waste. Their findings showed that the Fe recovery was 98.1% under optimized conditions. Tang et al. [126] reported the recovery of iron from iron ore tailings. They used it to develop a concrete composite mixture. They investigated the impacts of different parameters upon the extraction of ferrous particles. Up to 83.86 wt% of iron was recovered from a feed iron grade of 12.61 wt%. Zhang et al. [127] attempted to recover iron from the waste slag of pyrite processing using a reduction roasting magnetic separation method. The iron content of the concentrate was initially 57%, of which 87% was extracted using the aforementioned method. It should also be noted that, through further treatment using chlorinated segregation–magnetic separation, the iron content in the slag was increased to 83%.

In the study carried out by Wang Yu et al. [128], the co-precipitation and magnetic separation methods were adopted with the aim of recovering iron from waste ferrous sulphate. They investigated the impacts of various reaction parameters on the iron recovery, and also examined the impacts of milling time and magnetic induction intensity on the separation of magnetic particles. The mixed magnetic particles were wet-milled for 20 min before magnetic separation. The grade and recovery rate of iron in the magnetic concentrate drastically increased from 51.41% to 62.05%, and from 84.15% to 85.35%, respectively.

### 2.4. Gypsum Waste (CaSO_4_⋅ 2H_2_O)

**Figure 7:**
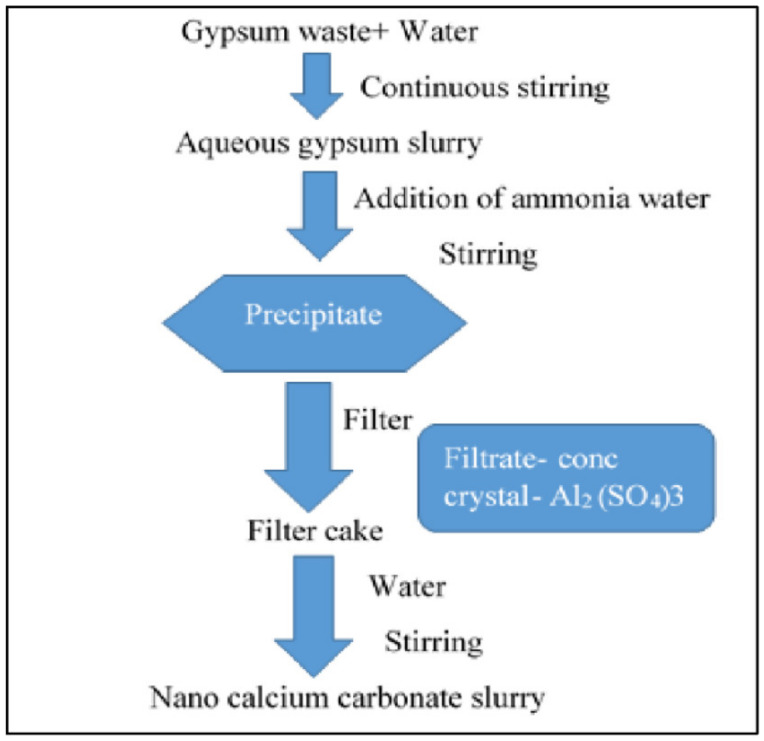
Steps involved in the synthesis of calcium carbonate nanoparticles from gypsum waste, adapted from Yadav et al., open access journal Applied Sciences, 2021 [129].

Gypsum waste is a byproduct of the gypsum industry. It is widely used in dental applications, with its disposal representing a potential threat to the environment [129]. There is a possibility that when disposing of gypsum waste in landfill, there might be a reaction between the gypsum and biodegradable waste, which may produce poisonous and odorous hydrogen sulfide gas [130,131]. Gypsum waste can be processed for the recovery of CaS, which ultimately changes to calcium carbonate. A schematic diagram of the process of recovering calcium carbonate from gypsum waste is shown below in Figure 7, as reported by Yadav et al., 2021 [129].

Beer et al. [132] attempted to synthesize calcium carbonate nanoparticles from gypsum waste, producing elemental sulfur as a byproduct. In this process, the first step was the thermal reduction of the gypsum waste into calcium sulfide (CaS), followed by its direct aqueous carbonation, yielding low-grade carbonate products (i.e., 99 mass% as CaCO_3_) or precipitated calcium carbonate (PCC). The carbonate product was found to be predominantly composed of calcite (99.5%) with only 0.5% quartz. Calcite was the only CaCO_3_ polymorph obtained in the experimental process, while no vaterite or aragonite was found [133]. In addition, Mulopo and Radebe [129] investigated the batch recovery of calcium carbonate from gypsum waste slurry using sodium carbonate under ambient conditions. The results were applied to the pre-treatment of acid mine drainage (AMD) from coal mines. US patent no 2013/0288887 A1 reports a simple, cost-effective, and novel method for the recovery of nano-calcium carbonate from gypsum waste slurry [134]. Okumura et al. [135] extracted calcium oxide particles from the gypsum waste using reductive decomposition in a CO-CO_2_-N_2_ atmosphere. They also investigated the reductive decomposition of spent CaSO_4_ using a packed-bed reactor. The CaSO_4_ was used for the production of calcium oxide. Ramachandran and Maniam [136] reported a two-step method comprising chemical and thermal reactions for the regeneration of calcium oxide from gypsum waste. The chemical changes and confirmation of the formation of calcium oxide were determined using XRD. Mbhele et al. [137] attempted to recover sulphur from gypsum waste using the following sequence of steps: (1) reduction of gypsum to CaS_2_ (2) stripping of the sulphide with carbon dioxide gas, and finally (3) the production of S. 

### 2.5. Agricultural Waste

A great deal of solid agricultural waste is generated each year, presenting a major challenge for disposal processes, as it creates a foul odor and attracts pests, resulting in health issues for living beings. Some of the most commonly generated types of agricultural waste include rice husks [138], rice straw [139], wheat husks and straw, coconut shells [140], sugarcane bagasse [141], corn cobs, and almond shells. These types of agricultural waste are rich sources of carbon, silica, calcium, and other trace elements like Fe, Al, etc. Even today, only a small fraction of such agricultural waste is applied as a fuel, additive, and filler in the construction industry. A major fraction of agricultural waste is disposed of using three main techniques, thermal treatment, landfill, and decomposition, and these have been reported to negatively affect the environment [26]. For example, the thermal treatment of waste results in the release of numerous noxious gases like CO_2_, CO, Cn Hm, SOx, NOx, ash, etc., many of which are classified as greenhouse gases. As a result, many scholars have raised objections over combustion as a method of agricultural waste disposal. 

Agricultural waste such as sugarcane bagasse and rice husks constitute a rich source of silica. Both rice husks and sugarcane bagasse can act as potential candidates for silica extraction. The silica synthesized from such wastes can provide an alternative, renewable source, minimizing the pollution resulting from such materials. Meanwhile, coconut shell husks are a rich source of calcium [26]. The possibility of recovering of such valuable minerals from waste not only provides alternative precursor materials, it also provides an environmentally friendly, cost-effective approach to the problem. The use of such agricultural waste also minimizes environmental pollution. All of these waste products are biological materials; thus, they are rich sources of carbons that can be used for the synthesis of activated carbons or biochars. 

There are certain plants that possess a high accumulation of silica in their leaves, stems, fruits, etc. The silica is taken up from the soil by the roots of the plants and distributed to the other plant parts. In plants, silica is mainly present as silicon. Silica can potentially be found in the solid waste used extensively in the industrial sector. Two factors, namely, the availability and quantity of silicon in the soil, affect silica deposition in agricultural residues [142]. Several plants consist of silica, includingwheat, rice, sunflowers, corn, and bamboo [143]. In general, silica can be extracted from leaves, stems, and other parts of a plant with a yield of between 0.1 and 10 wt%. On the other hand, the quantity of silica in agricultural residues is dependent on the season, species, maturity, and geographical characteristics of the given farm.

#### 2.5.1. Rice Husks (RH) and the Recovery of Silica

Rice husks are agricultural waste products that are produced during processing. During the hulling process, rice husks are obtained, which are mainly used as fodder for cattle. Rice husks and straw are major agricultural products, with an annual global production as high as one million tons. These waste products are common in rice-producing countries like India, Vietnam, and Japan. RH are rich is silica, but at the same time they also contain organic compounds that may interfere with the final purity of the silica. Therefore, RH have to first be calcinated at high temperature (400–1000 °C) in a muffle furnace. Furthermore, they have to be washed with phosphoric acid, which will remove the organic content. Once the RH ash is free from organic content, it is dried and treated with 4–16 M NaOH, along with 90–95 °C, for 60–90 min along with continuous stirring. NaOH will react with NaOH and will form sodium silicate, which will be further treated with 1–2 N HCl, resulting in silica gel. This silica gel is further washed and dried to obtain pure silica or nanosilica. Some authors have also reported calcination at 400–600 °C for 2–6 h in order to obtain nanosilica with the desired shaped [144].

#### 2.5.2. Sugarcane Bagasse and the Recovery of Silica

Similarly, sugarcane bagasse is also one of the major byproducts of the sugarcane industry, and is produced after the extraction of sugarcane juice. Sugarcane bagasse is a rich source of carbohydrates and other minerals besides silica. It is also produced in huge quantities in sugar-producing countries. Sugarcane bagasse (SB) is considered to be a non-biodegradable solid material, and primarily consists of crystalline silica [145]. A major problem in the sugarcane industry is how to dispose of SB. At present, it is used in the production of ceramic tiles, soil fertilizers, and fodder in some parts of the world [146]. In comparison with other agricultural residues, sugarcane bagasse ash (SBA) possesses a very high quantity of silica. Table 2 presents the complete elemental composition of SBA. Several parameters affect the quantity of silica extractable from bagasse, including the nature of the soil, the surrounding environment, the harvesting process, and the time of harvesting.

Drummond et al. [147] reported silica extraction from different preparations of SBA by performing natural burning (SBA-NB) and laboratory burning (SBA-LP) at 700 °C for two hours using muffle furnace, which was followed by alkaline extraction. The experiments revealed that silica extraction of about 94.47% was achieved using natural burning, and 96.93% of the silica was obtained using laboratory burning. In another project, Harish et al. [148] attempted to recover silica from SBA and silica fumes as low-cost precursors for the synthesis of silica gel using a sodium hydroxide-based alkali treatment method. Norsuraya et al. [149] reported the synthesis of Santa Barbara Amorphous-15 from SBA. XRF revealed that raw sugarcane ash contained 53.10% silica, while acid-treated ash contained 88.13% silica.

Channoy et al. [141] conducted a study aiming to synthesize silica gel from SBA by treating the ash with 1.5, 2, and 2.5 N NaOH. The particle size of the obtained silica gel varied, with values of 120, 100, and 80 nm, respectively, as confirmed by SEM. SEM revealed that as the concentration of NaOH increased, the particle size decreased. Rovani et al. [150] made use of SBA waste to synthesize highly pure silica with a high adsorption capacity. The synthesized silica nanoparticles were characterized by FTIR, SEM, TEM, XRD, ICP, etc., and it was found that the particle size was 20 nm and the purity was about 88%.

To obtain silica from SBA, several steps are needed: washing of the collected SB, shredding it into small pieces, pyrolysis at high temperature, alkali treatment of ash, acidic treatment of the sodium silicate leachate, formation of the silica gel, washing, precipitation, and calcination.

#### 2.5.3. Coconut Shells

**Figure 8:**
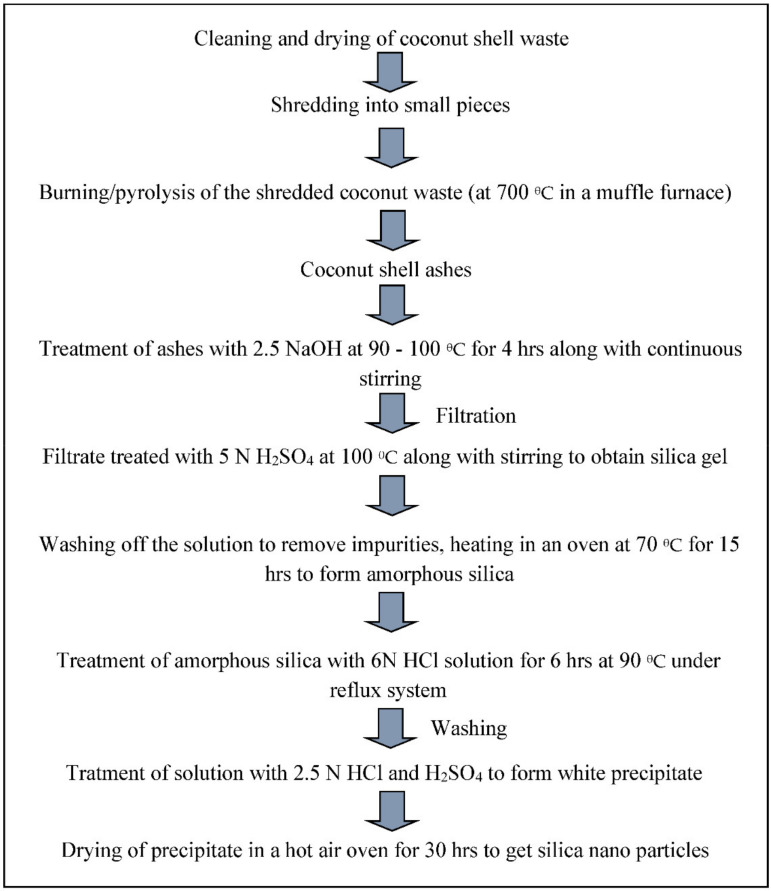
General method for the synthesis of silica from coconut shell waste.

Coconut (Cocos nucifera) is the major plantation crop of coastal countries such as African countries [151]. A large number of coconuts are produced around the world, and these are mainly used for food and cosmetics. The soft part of the fruit is consumed, while the coir and shell are left behind as waste. Coir is used to make mattresses, while the shell is mainly disposed of into the environment. However, in African countries, coconut waste is conventionally used as a source of fuel and applied in burning processes [152]. Nowadays, with recent research advances, coconut shell waste can be used as a source of activated carbon and/or other value-added minerals. Coconut shell has high silica content and can act as a potential candidate as a source of silica and activated carbon. The use of coconut shell as a source of both silica and activated carbon presents cost-effective green synthesis strategy. Coconut shell can act as another substance for the synthesis of silica from renewable sources. Roughly 33–35% of a coconut is composed of husk, playing the role of the mesocarp of the fruit. Nowadays, coconut husk is used as a favorable source of fuel for coconut processing and domestic fuel, and as a fiber source for the production of mats, ropes, etc. [153]. The extraction of silica from agricultural wastes involves three sequential steps: acid leaching, mixing alkaline treatment, and precipitation with acid [154]. A schematic diagram is shown in Figure 8. The chemical composition of coconut shell is given below in Table 3. It mainly contains calcium oxide, followed by silica, along with traces of Al, K, Fe, and P, in the form of either oxides or chlorides.

##### Synthesis of Carbon Nanotubes from Coconut Shell Husk Ash

Carbon nanotubes have gained considerable attention from scientific fields of study such as medicine, drug delivery, photo dynamic therapy, and environmental cleanup. They have numerous advantages over other nanoparticles due to their high mechanical properties, high tensile strength, high electrical conductivity, high aspect ratio, high thermal conductivity, and ultra-light weight. Generally, these nanotubes are synthesized using the physical vapor deposition method or the chemical vapor deposition method; both of these methods are costly and energy intensive. As a result, the synthesis of carbon nanotubes from carbon-rich waste materials like coconut shell husk ash is an economical and environmentally friendly approach. There are several approaches in which coconut shell has been used for the synthesis of carbon nanotubes.

Anuar et al. [153] reported the synthesis of silica nanoparticles from coconut shell ash. In their study, the coconut husks were burned under different temperatures, and were analyzed using XRF to identify their elemental composition. The composition of silica varies from 8–11% in the husk ash. The husks and silica were characterized by SEM-EDS, XRD for confirmation and to determine the optoelectronic properties of the silica. In another project, Sivasubramanian and Sravanthi (2015) attempted to synthesize silica nanoparticles from coconut shell ash using the NaOH-based alkali treatment method. Ash was treated with 2.5 N sodium hydroxide to obtain sodium silicate. Finally, silica was formed by treatment with HCl, and was confirmed by SEM-EDS, FTIR, TEM, and XRD [155]. Melati and Hidayati [156] reported the synthesis of multi-walled carbon nanotubes from coconut shell in two steps. First, the coconut shell was activated by treating it at 500–600 °C, and it was subsequently converted into carbon nanotubes by applying pyrolysis and a wet CVD process. The characterization of the nanotubes revealed the properties of the MWCNTs. It was used for the detection of cancer in mammalian cell lines.

In the study conducted by Adewumi et al. [157], carbon nanospheres were synthesized from low-cost coconut fibers in three sequential steps: pyrolyzation, physical activation, and ethanol vapor treatment. The analysis of the samples revealed that the spherical-shaped particles had a diameter of 30–150 nm. Hakim et al. [158] applied an easy, environmentally friendly approach called the one-step water-assisted (quenching) synthesis method to obtain carbon nanotubes using coconut shell husk ash. The chemical and physical properties of the carbon nanotubes were analyzed using sophisticated instruments, and it was found that the average diameter was 123 nm; the nanotubes were finally applied for the remediation of Pb^2+^ ions from wastewater. Araga and Sharma [158] synthesized PECV-assisted multiwalled carbon nanotubes (MWCNTs) over coconut shell-derived charcoal pyrolyzed at 900 °C in a process with only a single step. They used the mineral content in the source material as the catalyst for carbon nanotube (CNT) growth.

##### Corn Cobs as a Source of Activated Carbon

Maize, or corn (*Zea mays*), is a popular cereal crop cultivated in many parts of the world [159]. During the processing and production of corn, several waste products are generated, including corn cobs and corn husks [160]. Corn cob waste is a rich source of carbon, and can act as a potential candidate for reparing carbon with ultra-high specific surface area [161]. On the other hand, there are various waste materials that can be applied as activated carbon sources, for instance, date and palm waste, coconut shell waste, corn stalks, and corn cobs. Corn cob is most preferable source of activated carbon, as it is produced in huge amounts around the world. Corn cobs are agricultural waste materials produced in abundance around the world. After extracting the corn, the major fraction of the corn is disposed of as waste, although it is actually a rich source of various minerals. The cob is mainly made up of carbons; therefore, it can be used as a source of biochars or activated carbon. 

Activated carbon (AC) refers to carbonaceous materials that possess an internal surface area (that is extremely developed) as well as porosity [162]. The large surface area (an area between 250 m^2^/g and 2000 m^2^/g) offers a significant ability to adsorb chemicals from liquids/gases, and permit application as a versatile adsorbent under various conditions. AC has been widely used to produce adsorbents, supporting materials, textiles, fabrics, animal foods, etc. [163]. AC is known to be an effective material as a result of its low density, well-developed porosity, accessibility, chemical stability, and low cost [164]. In the last few years, a major emphasis has been placed on the development of AC with ultra-high specific surface area from both renewable and non-renewable sources using chemical approaches or with the use of chemical vapor deposition (CVD). In the process of water treatment, a substantial amount of AC is used for the purpose of removing organic and other compounds that could change the water odor and taste [165].

**Figure 9:**
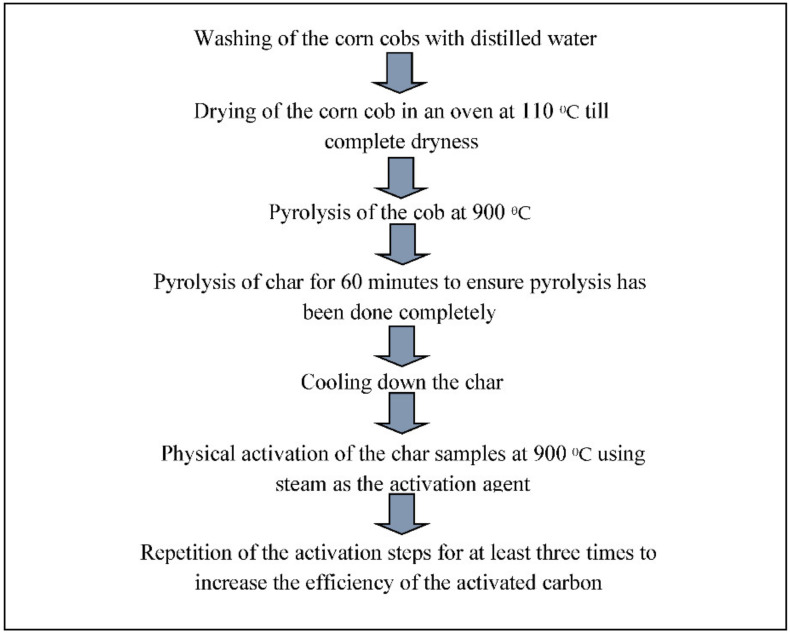
Schematic diagram for the formation of activated carbon from corn cob.

Tsai et al. [166] reported the synthesis of AC from corn cob waste through treatment with different physical and chemical activators such as NaOH, KOH, K_2_CO_3_, and CO_2_. They studied the effect of impregnation time, impregnation ratio, activation temperature, and soaking time on carbon dioxide. The surface area of the AC was analyzed using the BET analyzer. The total pore volume and BET surface area were roughly 1.0 cm^3^/g and 2000 m^2^/g, respectively. The findings of this research demonstrate that corn cob activation with KOH/K_2_CO_3_ and CO_2_ is able to appropriately prepare large-surface-area ACs. Furthermore, Sai et al. [167] attempted to activate corn cob using potassium salts, and subsequently gasified it with carbon dioxide. The obtained AC had a large surface area, as measured by BET. In the study carried out by Kazmierczak et al. [168], AC was developed from corn cob by activating it using chemical and physical methods. They further studied the sorption properties of the activated carbon. The final product consisted of microporous activated carbon with a high surface area, varying from m 337 to 1213 m^2^/g, and showing diverse acid–base characteristics on the surface [169,170]. It was also assessed for the adsorption of different materials from the aqueous solutions. Figure 9 shows the schematic diagram for the formation of activated carbon from corn cob.

## 3. Domestic Waste

Domestic waste refers to the materials produced in houses, mainly kitchens. There are several wastes produced in houses that offer potential for the recovery of value-added minerals. Calcium carbonate is a highly important particle that is used in every aspect of our life, as well as in a number of different industries [171]. Calcium carbonates are alkaline earth materials [172] that are present in our environment and which are widely applied in industries like papers [173], paints [174], coating agents, cosmetics [175,176], pharmaceuticals and medicine [177], agriculture, automobiles and textiles [178], and reinforcements, fillers, bio-nanocomposites and bio-ceramics in dentistry [178,179]. 

Calcium carbonates have excellent properties, including biodegradability, biocompatibility, pH sensitivity, safety, and cost-effectiveness, and they exhibit polymorphicity [179]. They are very lightweight; thus, they can be used for making lightweight materials. Calcium carbonate exists in three polymorphs in nature, i.e., calcite, aragonite, and vaterite [180]. All these three polymorphs vary with respect to their thermodynamic stability and morphology. Among them, calcite is thermodynamically the most stable, while aragonite shows an intermediate stability, and vaterite (μ-CaCO_3_) is the least stable polymorph, due to which it is less commonly present in nature. Vaterite can be rapidly transformed into the aragonite and calcite in aqueous solution. It has been experimentally proved that vaterite can transform to aragonite in 60 min at 60 °C and to calcite in 24 h at room temperature [181]. While calcite is rhombohedral in structure, aragonite is spindle- or needle-shaped, while vaterite is octahedral [182]. There are several waste materials that are rich sources of calcium oxide and calcium carbonates, including incense sticks, eggshells, cockle shell waste [183], and gypsum waste; however, the following sections only discuss domestic waste, i.e., incense stick ash and eggshell waste.

### 3.1. Incense Stick Ash (ISA)

**Figure 10:**
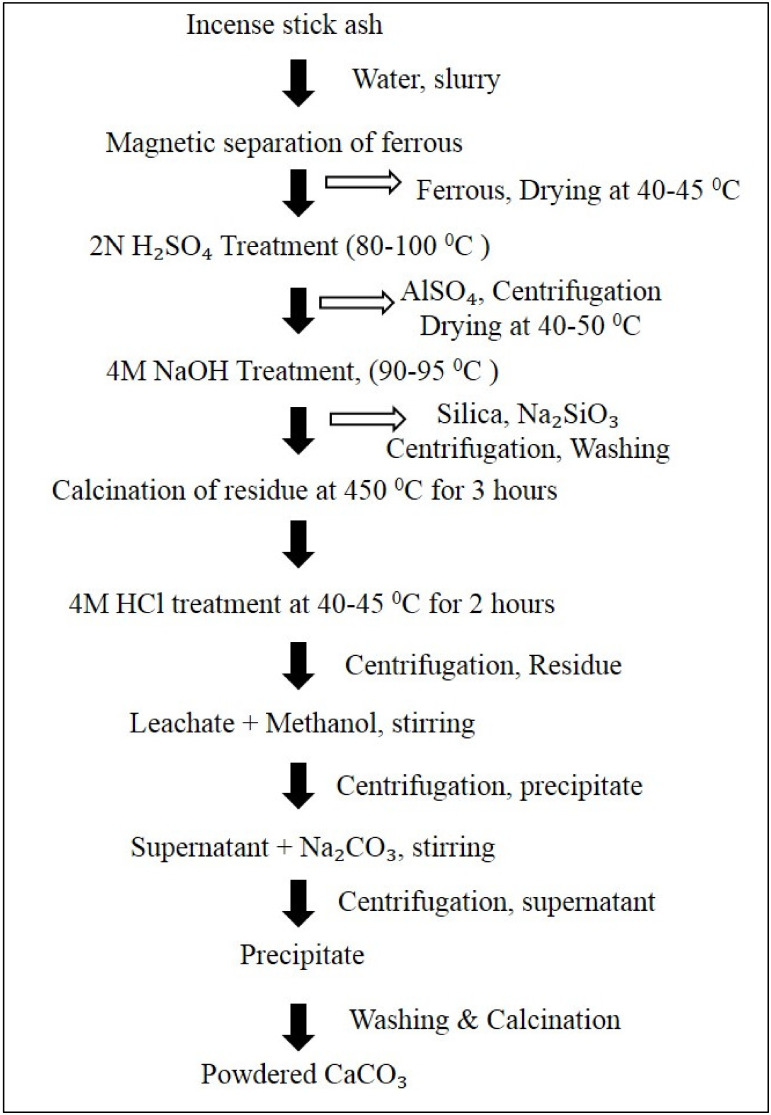
Flow chart for the synthesis of CaCO_3_ from incense stick ash, adopted from Yadav et al., open access journal Applied Sciences, 2021 [129].

Incense stick ash is one of the most unexplored products, and are generally produced at religious places, i.e., temples, churches, mosques, etc. The burning of incense sticks is a ritual practiced in every religion. However, in South-Asian countries and zones, e.g., China, Taiwan, India, and Japan, large amounts of incense sticks are consumed [184,185]. Incense sticks are cylindrical in shape, and are fragranced and intended to be burned in order to purify the air [186]. After the burning of incense sticks, the ash is left behind in the form of a residue, which is mainly disposed of in rivers and other water bodies, as can be frequently observed in India, where this practice is considered to be holy. Moreover, due to its sacred value in Hinduism, incense is even applied on the forehead or eaten as Prasad. These incense sticks mainly contain calcium oxide, silica, ferrous, alumina, rutile, Mg, and traces of oxides of K, Na, and Mn. The sticks contain 45–60% calcium oxides or carbonates, 10–20% silica, and 5–7% Mg, with less than 5% consisting of other materials. The disposal of incense stick ash into water pollutes it by increasing the concentration of Ca and Mg, ultimately increasing the water hardness. These two elements also play a role in increasing the pH of water by forming precipitates of hydroxides in it. Due to the high calcium content, incense stick ash can act as a potential source material for the recovery of calcium oxides and carbonates. The complete schematic diagram for the synthesis of calcium carbonate from incense stick ash is given below in Figure 10.

### 3.2. Eggshell Waste

Eggshells are another waste product produced by houses and industry, and are still classified as a byproduct of the poultry industry. Approximately one million tons of eggshell are generated per year globally. Currently, eggshell waste is dumped into landfills. Such practices may lead to the deterioration of agricultural land [187]. This waste is mainly made up of calcium oxides that are meant to provide safety to growing chicks. Due to their high calcium content (composition shown in Table 4), eggshells can be used as a potential alternative source of calcium oxide or carbonates [188,189].

The recovery of calcium oxides and carbonates from incense stick ash and poultry is easy, economical, and environmental friendly. Recovery from such wastes focuses on the use of renewable sources without affecting our natural resources. Consequently, it also minimizes pollution in the form of solid waste. Hassan et al. [190,191] reported the synthesis of CaCO_3_ NPs from chicken eggshell waste, which involved the following steps: cleaning and size reduction of eggshells, followed by surface modification using the sonochemical method to achieve enhanced dispersion [190,191]. In another study, Hariharan et al. [192] attempted to perform the synthesis of calcite nanoparticles from eggshell waste using gelatin. Chicken eggshells were used to obtain the calcite polymorph of calcium carbonate using gelatin by means of the precipitation method. Nanocalcite was confirmed using FTIR, XRD, UV-Visible spectroscopy, and SEM. The particles were identified as calcite polymorphs with a particle size of 25 nm. The results obtained in the FTIR experiments confirmed the creation of calcite, with characteristic absorption bands being observed at 712, 876, and 1410 cm^−1^, corresponding to the bending and stretching vibrations of CO_3_^2−^ ions. A comparison was also made between the obtained results and calcium carbonate synthesized with no gelatin.

Calcium oxides are currently produced at commercial levels from calcium-based precursor materials like calcium nitrate [193], calcium hydroxide [194], calcium sulphate, and calcium chloride [193] using techniques such as thermal decomposition [194], microwave irradiation [195], sol–gel [196], co-precipitation [197,198], hydrogen plasma–metal reaction [199], and sonochemical synthesis [191,200]. However, the use of the above-mentioned calcium precursors for the synthesis of calcium oxide nanoparticles using the techniques described above makes the whole process costly and energy intensive, and also requires the use of hazardous chemicals. As a result, for the synthesis of calcium oxide nanoparticles, there is a need to switch to green methods. Some of the most common calcium-rich waste materials are cockle shells [201], eggshells [202], gypsum waste, and incense stick ash. Among these, incense stick ash is the most underestimated calcium-rich waste material, and is produced abundantly at religious places and in houses. Such materials can act as potential substitutes for various calcium precursors used at commercial level [203,204]. 

Tangboriboon et al. [205] synthesized calcium oxide nanoparticles from duck eggshell waste using the calcination technique and analyzed their properties. The duck eggshells and the calcined eggshells were analyzed using FTIR, STA, XRD, XRF, TEM, BET, a particle size analyzer, and an impedance analyzer. The microscopy revealed the good dispersion quality of the calcium oxide nanoparticles, which had a spherical shape, with a ceramic yield of 53%. In another study, Jirimali et al. [206] reported the synthesis of calcium oxide and hydroxyapaptite using eggshell for the development of LLDPE Polymer Nanocomposite [206]. Mohadi et al. [202] synthesized the calcium oxide nanoparticles from the chicken eggshell waste by calcination of the shells at different temperatures ranging from 600 to 1000 °C. The synthesized calcium oxide nanoparticles were analyzed using FTIR, SEM-EDS, XRD, and BET. The calcium oxides were porous in nature, with pore sizes of 6.6 nm, meaning they could be classified as mesoporous, and with a surface area of 68 m^2^/g [202].

**Figure 11:**
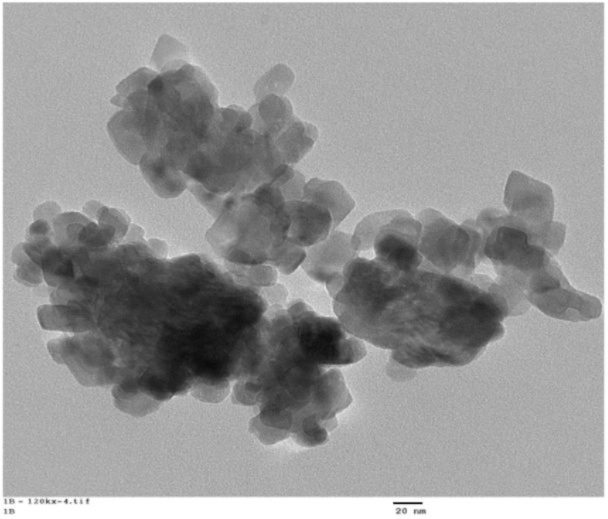
TEM image of calcium oxide nanoparticles obtained from eggshell waste adapted from (Render et al. [204]).

A typical TEM image of calcium oxide nanoparticles obtained from eggshell waste is shown in Figure 11 [204].

There are several reports of the synthesis of composite materials and fertilizers from plastic waste; however, research conducted in this field is very limited. A lot of work needs to be performed in this field in future, since plastics constitute a major global problem.

## 4. Conclusions

Waste management mainly addresses two subjects: resource recovery and final disposal. Individuals worldwide earn revenue from each stage, through the recovery of reusable materials and, to a lesser extent, the conversion of waste to energy. Turning waste into wealth not only makes sensible environmental sense, but also turns “trash” into “cash”. Agricultural waste materials are mainly organic in nature; they are biodegradable, and can be used for the development of carbon-based materials and activated carbon. The burning of agricultural waste leads to the pollution of the air. In recent decades, waste management and technology awareness programs have successfully transformed hazardous materials (e.g., CFA and red mud) into useable value-added minerals. Both of these materials have been applied in the fields of ceramics, construction materials, and metallurgy, and have proved to be highly valuable. Poultry waste and domestic waste have been also been found to be important precursors of carbon and calcium oxides. The ash from incense sticks is among the emerging domestic byproducts produced at religious places, and have proved to be a valuable source of calcium oxide. All of these waste byproducts have gained importance with the advent and increasing significance of renewable energy sources. The recovery of minerals from such wastes is an economical and environmentally friendly method. Such materials act as alternative precursors, reducing the burden on industry. The use of such wastes reduces pollution at minimum cost, while developing materials and generating revenue.

## Figures and Tables

**Table 1:** Elemental composition of red mud.

Composition	Percentage
Fe_2_O_3_	30–60%
Al_2_O_3_	10–20%
SiO_2_	3–50%
Na_2_O	2–10%
CaO	2–8%
TiO_2_	2–5%

**Table 2:** Elemental composition of sugarcane bagasse ash (SBA).

Elements	Raw Sample	Sample after Acid Treatment
SiO_2_	53.10	88.13
MgO	20.72	3.04
CaO	3.77	0.57
SO_3_	11.20	4.69
P_2_O_5_	7.36	1.15
K_2_O	1.26	0.50

**Table 3:** Elemental composition of coconut husk ash (CHA).

Elements	Composition (%)
SiO_2_	8–12
CaO	27–31.5
K_2_O	17–20
Al_2_O_3_	0.3–0.8
SO_3_	2–3.5
Fe_2_O_3_	0.3–1.0
P_2_O_5_	0.05–0.3
Cl	35–38

**Table 4:** The chemical composition of chicken eggshells.

Chemical Elements	Concentration (mg/L)
Ca	2296–2304
Mg	849–852
Na	33–35
K	16–19
Fe	1.01–1.43
Zn	0.95–1.03
Cu	0.062–0.064

## Data Availability

Not applicable.
